# Effectiveness of Adalimumab in Non-radiographic Axial Spondyloarthritis: Evaluation of Clinical and Magnetic Resonance Imaging Outcomes in a Monocentric Cohort

**DOI:** 10.1097/MD.0000000000001170

**Published:** 2015-07-31

**Authors:** Luca Cantarini, Marta Fabbroni, Rosaria Talarico, Luisa Costa, Francesco Caso, Gian Luca Cuneo, Bruno Frediani, Gabriele Faralli, Antonio Vitale, Maria Giuseppina Brizi, Luciano Sabadini, Mauro Galeazzi

**Affiliations:** From the Rheumatology Unit (LC, MF, BF, AV, MGB, MG), Department of Medical Sciences, Surgery and Neurosciences, University of Siena, Siena; Rheumatology Unit (RT), Department of Clinical and Experimental Medicine, University of Pisa, Pisa; Rheumatology Research Unit (LC), Department of Clinical and Experimental Medicine, University Federico II, Naples; Rheumatology Unit (FC), Department of Medicine DIMED, University of Padua, Padua; Neuroradiology Unit (GLC), S. Donato Hospital, Cardiovascular and Neurologic Department, Arezzo; Radiology Unit (GF), S. Donato Hospital, Department of Diagnostic and Clinical Pathology, Arezzo; and Rheumatology Unit (LS), Department of Internal Medicine, Arezzo, Italy.

## Abstract

The primary aim of the study was to evaluate the long-term effectiveness of adalimumab (ADA) in a cohort of non-radiographic axial spondyloarthritis (nr-axSpA), and the secondary aims were to identify predictive factors of response and evaluate radiological progression.

We evaluated 37 patients (male/female: 12/25; mean age 49 ± 14; mean disease duration: 6.3 ± 5.8) with active nr-axSpA (Assessment of SpondyloArthritis International Society criteria), despite the treatment with ≥1 nonsteroidal anti-inflammatory drug for at least 3 months, initiating the treatment with ADA 40 mg every other week. Patients were treated for 24 months, and evaluated at baseline, 6, 12, and 24 months. Outcome measures included Ankylosing Spondylitis Disease Activity Score, Bath Ankylosing Spondylitis Disease Activity Index (BASDAI), and Bath Ankylosing Spondylitis Functional Index. Radiograph of the spine and sacroiliac joints and magnetic resonance of the sacroiliac joints were performed at baseline and according to the standard of assessment for the disease.

The proportion of patients that achieved a BASDAI50 response at 6, 12 and 24 months was 51.3%, 70.3%, and 76.8%, respectively. Treatment was well tolerated with no unexpected adverse events and/or serious adverse events. All patients remained on treatment for 2 years, with a good compliance. We did not identify any predictive factor of response to therapy. Moreover, modified Stoke Ankylosing Spondylitis Spine Score and Spondyloarthritis Research Consortium of Canada scores showed a trend of improvement during the study period.

ADA was effective on clinical and radiological outcomes at 2-year follow-up; thus, early treatment with ADA may prevent radiographic damage and be associated with low disease activity or remission. Moreover, data from this cohort study have confirmed safety and tolerability profile of ADA in nr-axSpA in the long term.

## INTRODUCTION

Several data suggest that magnetic resonance imaging (MRI) can show abnormalities even many years before the first radiographic change in spondyloarthritis (SpA).^1^ Therefore, the classification criteria developed by the Assessment of SpondyloArthritis International Society (ASAS)^2–4^ take advantage of MRI findings to identify patients who have axial SpA without radiographic changes (non-radiographic axial SpA: nr-axSpA). Although the existence of nr-axSpA is widely accepted, it is not so clear whether nr-axSpA can be included in the axial SpA family or it may represent a different entity. Globally, growing evidence suggests that nr-axSpA is very similar from ankylosing spondylitis (AS), and this is mainly because of genetic background, clinical patterns, disease activity, or disease burden.^5^ The major difference seems to be associated with the absence of ossification in nr-axSpA; however, so far, it is unknown how long is the probability of remaining free of radiographic changes throughout the course of the disease. The main advantage of recognizing the entity of nr-axSpA is mainly focused on starting treatment options in the window of opportunity of that early stage; on the other side, the risk of an overtreatment in those patients who will not present radiographic changes should be accurately balanced.

Literature data have shown that drugs such as TNF (Tumor Necrosis Factor) antagonists seem useful in controlling the symptoms and improving quality of life in nr-axSpA. Specifically, the ABILITY trial, a randomized, double-blind, placebo-controlled trial,^6^ compared the efficacy of adalimumab (ADA) versus placebo in nr-axSpA after 12 weeks of treatment, showing a sustained efficacy of ADA over the longer term. However, because the trial evaluated only short-term efficacy, it should be justified exploring whether a longer treatment period with ADA might have a different clinical and radiological impact. Indeed, data from observational studies are necessary to improve this knowledge.

Therefore, the present study aimed at evaluating the long-term effectiveness of ADA in a monocentric cohort of early nr-axSpA.

## PATIENTS AND METHODS

We evaluated 37 patients with early, active nr-axSpA, despite the treatment with ≥1 nonsteroidal anti-inflammatory drugs (NSAIDs) for at least 3 months, initiating the treatment with ADA 40 mg every other week.

The patients were consecutively included in this cohort study between 2007 and 2014. All but 2 patients [who started ADA before 2009, meeting the European Spondyloarthropathy Study Group (ESSG) criteria] met ASAS criteria for axSpA. Demographic profile of patient studied is shown in Table 1.

**TABLE 1:**
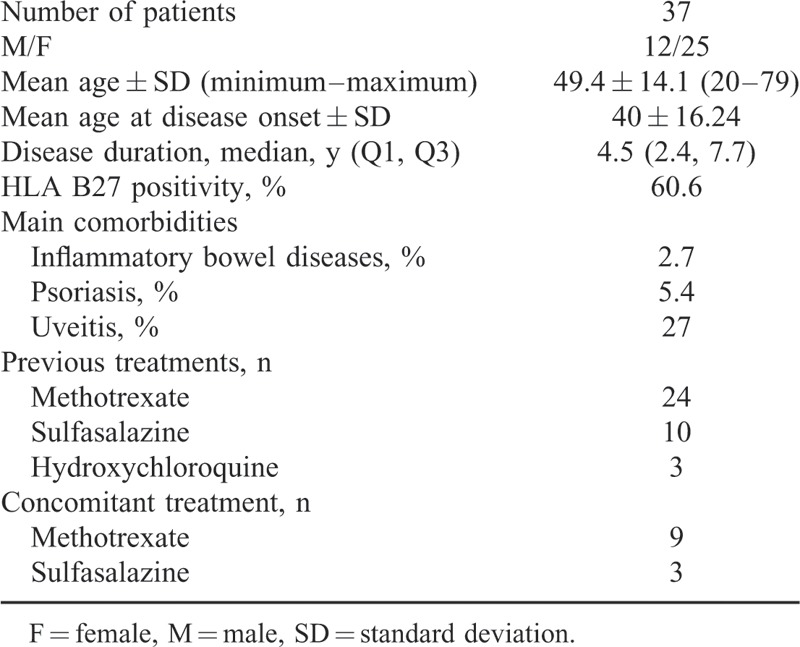
Demographic Profile of the Cohort Studied

The primary aim of the study was to evaluate the effectiveness of ADA in a cohort of early active nr-axSpA. The secondary aims were to identify predictive factors of response to therapy, evaluate radiological progression, and assess interobserver reliability between 2 radiologists reading magnetic resonance (MR) images of the sacroiliac joints performed at baseline and for posttreatment reassessment.

Patients were treated for at least 24 months, performing assessments at baseline, 6, 12, and 24 months. Epidemiological and clinical data were collected, including age of disease onset, disease duration, human leukocyte antigen (HLA) B27 positivity, and comorbidities (ie, psoriasis, inflammatory bowel diseases, and uveitis). Outcome measures included Ankylosing Spondylitis Disease Activity Score (ASDAS), Bath Ankylosing Spondylitis Disease Activity Index (BASDAI), and Bath Ankylosing Spondylitis Functional Index (BASFI). Radiograph (Rx) of the spine and sacroiliac joints and MR of the sacroiliac joints were performed at baseline and according to the standard of assessment for the disease (after at least 6 months for MRI and 12 months for Rx), and analyzed using the modified Stoke Ankylosing Spondylitis Spine Score (mSASSS) and the Spondyloarthritis Research Consortium of Canada (SPARCC) scoring method, respectively. Patients with radiographic sacroiliitis scored according to New York criteria,^7^ bilaterally score ≥2 or unilaterally score of 3 to 4, were excluded from the study. Decision on radiological involvement of the spine and sacroiliac joints were made upon consensus of 2 blinded readers. The study was approved by the local Ethical Committee of Azienda Ospedaliera Universitaria Senese, AOUS (Code: SPASI_2015 Study).

The primary endpoints were as follows:

1. the proportion of patients with a ≥50% improvement in BASDAI (BASDAI50 response) from baseline to 6, 12, and 24 months;
2. changes in ASDAS, BASDAI, and BASFI from baseline to 6, 12, and 24 months;
3. frequency of adverse events (AEs) and serious adverse events (SAEs) during the treatment period; and
4. treatment compliance evaluated by questionnaire regarding the number of medication not being taken.

The secondary endpoints included:

1. predictive factors of response to therapy, obtained investigating all the potential interactions between demographic and baseline characteristics and response to treatment;
2. spinal radiographs scored according to the mSASSS^8^;
3. MR sacroiliitis evaluated according SPARCC scoring system^9^; and
4. interobserver reliability between 2 blinded MR readers calculated using the proportion of agreement.

### Statistical Analysis

Quantitative data were expressed as mean ± standard deviation (SD) or median and interquartile ranges (Q1–Q3) for normally and nonnormally distributed data, respectively. Qualitative data are reported as numbers and percentages. Radiographic progression was determined by calculating the difference between the mean baseline and follow-up values. The continuous numbering/measurement scales were elaborated using nonparametric analysis of variance (Friedman test). Binary logistic regression analysis was used to identify factors associated with clinical response. Crude and adjusted odds ratios with 95% confidence intervals were also determined for each factors. For all analyses, statistical significance level was set at *P* values <0.05. Predictive power of the model was assessed by the area under the receiver operator characteristic curve (AUC-ROC). Possible overfitting was evaluated with the Hosmer–Lemeshow goodness-of-fit test. All statistical analyses were carried out using XLSTAT software.

## RESULTS

The proportion of patients that achieved a BASDAI50 response at 6, 12, and 24 months was 51.3%, 70.3%, and 76.8%, respectively. Table 2 shows the trend of mean ± SD of BASFI, BASDAI, and ASDAS from baseline to 6, 12, and 24 months. Moreover, the treatment was well tolerated with no unexpected AEs and/or SAEs. All patients remained on treatment for 2 years with good compliance. We did not identify any predictive factor of response to therapy; however, the presence of psoriasis and uveitis seems to correlate with a better outcome of response.

**TABLE 2:**
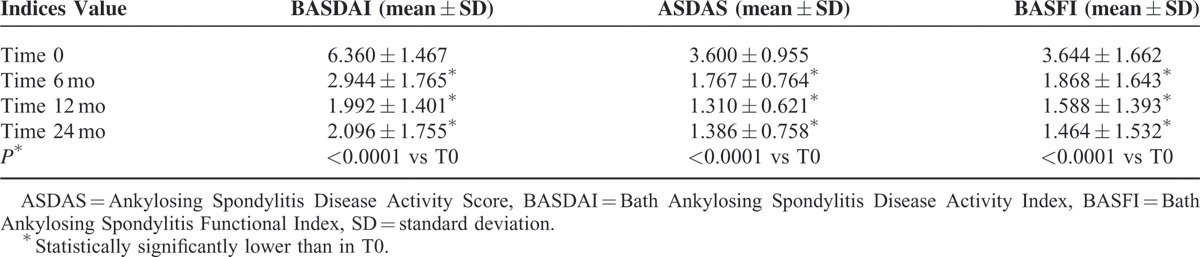
Trend of BASFI, BASDAI, and ASDAS During the Study Period

Regarding the radiological assessment, all patients performed MRI and Rx after a mean time of 18.7 (minimum: 6, maximum: 79) and 23.7 (minimum: 6, maximum: 76) months, respectively. As shown in Table 3, mSASSS and SPARCC scores showed a trend of improvement, although not statistically significant.

**TABLE 3:**
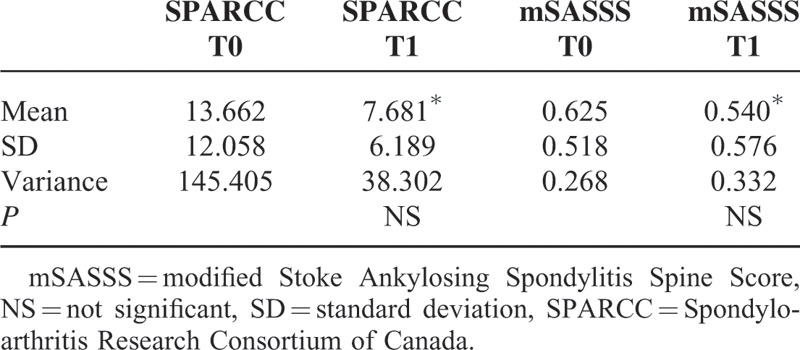
mSASSS and SPARCC Scores: Trend of Improvement at T0 and T1 (^∗^)

The proportion of agreement between the 2 MR readers was of 97.5% and 94.4% in the evaluation of images at baseline and post-treatment reassessment, respectively. The reliability between the 2 readers was highly significant (*P* < 0.0001).

## DISCUSSION

In the present article, we evaluated the long-term effectiveness of ADA in a cohort of nr-axSpA. Our results have shown that a significant proportion of patients achieved a good BASDAI50 response at each evaluation performed during the follow-up; a trend of improvement was also observed in the mean values of BASFI, BASDAI, and ASDAS from baseline during the overall study period. Similarly, a trend of change was detected in the mSASSS and SPARCC scores. The presence of psoriasis and uveitis showed a trend for a better response to treatment, but none of these factors was statistically significant. This was likely because of the small simple size. Globally, the treatment was well tolerated with no unexpected AEs and/or SAEs and with a satisfactory compliance, proved by the whole number of patients remained on treatment for 2 years. Furthermore, we evaluated interobserver agreement between 2 RM readers of sacroiliac changes in nr-axSpA patients, identifying a high interobserver reliability.

So far, a debatable issue is whether nr-axSpA represents an early disease stage of axial SpA or we should consider it as a distinct non-radiographic form of axial SpA.^5^ Literature data have extensively suggested that we can consider nr-axSpA as an important subgroup in the SpA spectrum. Specifically, longitudinal cohort studies have shown that patients with nr-axSpA do not necessarily progress to AS,^10,11^ and this is achievable substantially through the ASAS classification criteria. However, data from registries and clinical trials have shown that clinical symptoms and disease burden seem to be very similar between AS and nr-axSpA; thus, this issue may justify the obvious assumption that the same drugs will be effective in both conditions. It is well known that NSAIDs are considered the first-line therapy for patients with axial SpA,^12,13^ whereas traditional disease-modifying antirheumatic drugs such as methotrexate and sulfasalazine are not effective for the treatment of axial SpA.^14,15^ Anti-TNF agents are approved for the treatment of AS who continue to have active disease despite NSAIDs; therefore, in a scenario that did not provide an alternative treatment to NSAIDs for patients with nr-axSpA, the ABILITY-1 trial was developed.^6^ ABILITY-1 represented the first randomized controlled trial (RCT) to use the ASAS axial SpA criteria in classifying patients with nr-axSpA. The primary endpoint was represented by the percentage of patients achieving ASAS40 at week 12; efficacy assessments included BASDAI and ASDAS, and MRI was performed at baseline and week 12 using SPARCC index. Results from ABILITY-1 showed that ADA resulted effective in controlling disease activity, decreasing inflammation, and improving quality of life compared with placebo. Our results are in line with these previous findings regarding the overall efficacy of ADA; although our results are from a cohort study, the added values of our data are mainly related to the length of the observation (approximately 2 years) and the consequent information on the long term, both in terms of efficacy and safety. The main limitation of our study is the low sample size that did not allow us to correctly identify any predictive factor of response to therapy; in this regard, we are currently conducting a prospective study on a larger series to explore putative clinical, radiological, and serological biomarkers of early response to ADA and the others more frequently used TNF-α antagonist. Recently, an interesting meta-analysis was conducted to investigate the efficacy of TNF-α blockers versus placebo for the treatment of AS and nr-axSpA;^16^ the authors conducted a systematic literature search on RCTs investigating the efficacy of ADA, certolizumab, etanercept, golimumab, or infliximab in approved dosages in comparison with placebo. Twenty studies with data from 3096 patients were evaluated, of which 15 studies with AS patients, 4 with nr-axSpA patients, and 1 with both. Apparently, for AS patients, TNF-α blockers showed better efficacy than placebo for BASDAI, BASFI, and ASAS40 response compared with nr-axSpA patients; however, after adjustment for confounding factors, such as the year of publication of the study as a proxy for disease severity, no differences in the effect sizes between the AS and nr-axSpA trials were observed. The final analysis confirmed that anti-TNF-α agents improve disease activity and physical function in a clinically relevant way for patients with AS and nr-axSpA, showing no relevant differences in efficacy between patients with AS and nr-axSpA. To date, data on the reliability of detecting sacroiliac joints abnormalities in patients with recent onset inflammatory back pain are scarce. However, MR has been found to reliably detect inflammation and structural changes in sacroiliac joints in patients with early inflammatory back pain, with inflammation of subchondral region and bone marrow being the most frequently observed findings.^17,18^ In line with these findings, we identified a high interobserver agreement between MR readings, suggesting that 1 observer is enough to draw a conclusion on the presence of sacroiliac inflammatory involvement.

Further studies, both observational and experimental, are surely needed to improve our knowledge on the overall role of TNF-α antagonist on nr-axSpA, as well as the pragmatic use in the daily clinical management of nr-axSpA patients (ie, drug retention rates, reasons for treatment discontinuation, and appropriate assessment of comorbidities).^19,20^

## CONCLUSIONS

To sum up, our data have confirmed results from published studies, also in the long term. Even though more RCTs are needed in order to definitively confirm the evidence on the efficacy of TNF-α inhibitors in this indication, the results of the present study suggest that ADA has a positive benefit–risk profile in active nr-axSpA patients with inadequate response to NSAIDs.
